# Establishment of chicken muscle and adipogenic cell cultures for cultivated meat production

**DOI:** 10.3389/fnut.2025.1648935

**Published:** 2025-10-13

**Authors:** Vanessa Haach, Karine R. D. Silveira, Maíra de A. Peixoto, Ana Paula Passione e Sá, Vanessa Gressler, Vivian Feddern, Adriana M. G. Ibelli, Luciano Paulino Silva, Ana Paula Bastos

**Affiliations:** ^1^Embrapa Suínos e Aves, Concórdia, Brazil; ^2^Embrapa Clima Temperado, Pelotas, Brazil; ^3^Embrapa Pecuária Sudeste, São Carlos, Brazil; ^4^Embrapa Recursos Genéticos e Biotecnologia, Brasília, Brazil

**Keywords:** cultured meat, chicken cells, adipogenesis, myogenesis, biomass

## Abstract

**Introduction:**

Cultured meat seeks to replicate the sensory and nutritional attributes l of conventional meat by developing structured muscle tissue using cell culture. This study focuses on the culture of chicken embryonic and muscle-derived mesenchymal stem cells (MSCs) to derive muscle, and fat, optimizing conditions for differentiation and integration.

**Methods:**

We utilized monolayer and three-dimensional microcarrier-based cultures to produce muscle fibers and adipocytes while maintaining the extracellular matrix (ECM) integrity essential for tissue cohesion. Key pluripotency and myogenic markers (e.g., *cOCT4*, *cMYOD*, *cMYH1E*) were analyzed during differentiation, revealing dynamic gene expression patterns that underscore myogenesis.

**Results:**

Myoblast differentiation into mature myotubes demonstrated decreased *cPAX7* (−35%) and increased *cMYMK* (+67%), confirming lineage commitment and muscle fiber formation. Adipogenesis was induced in embryonic MSCs using food-grade lecithin, which activated *PPARγ*, *C/EBPα*, and *FABP4*,resulting in robust lipid droplet accumulation. To scale production, microcarriers facilitated cell proliferation, while transglutaminase-based stabilization enabled the formation of three-dimensional tissue structures comparable to native meat.

**Conclusion:**

Our findings highlight advances in culture protocols, genotypic and phenotypic expression analyses of multinucleated chicken muscle and adipocyte cells for cultured meat production.

## 1 Introduction

Over the past decade, the market has seen major shifts in consumer demand and product innovation for meat alternatives (1). Consequently, it is of paramount importance to sustain research efforts and develop alternative methods and proteins for the generation of novel products. Cellular agriculture is a novel sector that endeavors to eliminate the necessity for animal slaughter in order to offer a more sustainable alternative to conventional animal protein production. Among the developments in meat alternatives is the production of animal proteins from animal cell cultures or cultured meat (2). These advancements may contribute to addressing pressing challenges such as scarcity of food, climate change, animal welfare, and certain public health concerns, although these impacts remain dependent on broader social, economic, and regulatory factors (3–6). Nonetheless, this technique faces numerous technological obstacles.

The primary ingredient in cultured meat is animal cell lines, which must be cultivated under controlled conditions to proliferate and differentiate into muscle and fat tissues (7). An indispensable requirement for any bioprocess, particularly in the successful creation of cultured meat, is the availability of a cell line that exhibits consistent and replicable characteristics, along with the development of suitable culture systems (8). Nevertheless, the absence of availability to thoroughly characterized cell lines poses a substantial obstacle to the investigation of cultured meat. It is essential that the initial cell types exhibit a high proliferation rate or self-renewal capacity in order to attain sufficient quantities for the effective production of cultured meat. Additionally, these cells must be capable of differentiating into the completely developed cell types that constitute meat (9).

There are two notable strategies for establishing cell lines for cultured meat production: (1) utilizing a sample of the tissue of interest (primary cell sources), coupled with the isolation of progenitor cells residing in the muscle; and (2) employing pluripotent or multipotent stem cell sources, such as embryonic stem cells (ESCs) and induced pluripotent stem cells (iPSCs), which possess the capacity to differentiate into muscle-resident progenitor cells (10). Although stem cells, such as muscle stem cells and pluripotent stem cells, are widely employed as a cellular source for cultured meat, they are uncommon in the animal body and difficult to multiply on a large scale. Conversely, somatic cells, which compose the majority of the body, can be effectively transdifferentiated into muscle cells under specific circumstances.

The main cellular constituents of meat include skeletal myocytes, adipocytes, fibroblasts, and hematopoietic cell types (11). To optimize the production of cultured meat, it is crucial to determine the specific cultivation conditions that promote muscle cell proliferation and differentiation of satellite cells into myotubes and myofibers. These conditions should also preserve meat-like texture and flavor characteristics (12). Supplementary methodologies are required to isolate target cells and achieve additional purification from these preliminary cell extracts. Consequently, a variety of techniques are employed to purify cells, each of which possesses a distinct set of benefits and drawbacks (2). Current cell separation methods utilize surface proteins, differential adhesion, selective plating, genetic expression, and cell detachment (7). In addition, physical principles are employed to effectively isolate specific cells based on their phenotypic characteristics, including cell sorting (FACS) and capture using magnetic beads that are coated with cell-specific antibodies (13, 14).

The viability of the cultured meat sector largely depends on technological advancements in both industry and research. The majority of research conducted on cultured meat has mostly been on producing a product that mimics the appearance and texture of fresh meat by proliferating and differentiating muscle stem cells (15–17). However, producing sufficient samples of cultured meat for sensory panel testing remains a challenge (17), which makes it difficult to evaluate the technical flavor and texture attributes. Few studies have demonstrated evidence about the nutritional composition of cell lines, observing them as ingredients. In spite of these obstacles, it is possible to investigate and enhance muscle satellite cell culture techniques to guarantee that they exhibit flavor characteristics that are comparable to those of conventional meat. Our study was designed to resolve technological deficiencies and reinforce initiatives within the cultured meat industry. Our initiative was dedicated to the development of essential ingredients for the production of cultivated food products, specifically muscular and adipocyte chicken cell lines.

## 2 Materials and methods

This study was designed to isolate and characterize chicken embryonic stem cells (ESCs), mesenchymal stem cells (MSCs), and muscle satellite cells for applications in cultured meat. ESCs were isolated from stage X blastoderms, and MSCs and satellite cells were obtained from thoracic and hind limb muscles of 15-day-old specific-pathogen-free (SPF) chicken embryos. A total of 150 embryos were used across three independent experimental replicates (*n* = 3). For each replicate, cells from 10 to 20 embryos were pooled to reduce biological variability and ensure sufficient cell numbers for downstream analyses. This strategy was applied consistently for ESCs, MSCs, and satellite cells (Supplementary Figure S1). Myogenic and adipogenic differentiation assays were performed using cells at passages 3–8.

Fluorescence microscopy, RT-qPCR, and immunostaining were conducted to assess gene expression and phenotype at specific timepoints during differentiation. For each biological replicate, technical triplicates were included in RT-qPCR, lipid staining, and immunofluorescence assays. Microcarrier-based and spheroid cultures were also assessed in triplicate for morphological evaluation and viability analysis.

### 2.1 Chicken embryonic stem cells (blastoderm) isolation

Animals use was approved by the Animal Use Ethics Committee of Embrapa Suínos e Aves (protocol number 22/2022) and conducted in accordance with the Brazilian guidelines established by the National Council for the Control of Animal Experimentation (CONCEA) under Law No. 11.794/2008 and Decree No. 6.899/2009. Blastoderm cells at stage X of Eyal-Giladi and Kochav (EGK) (18) were isolated from unincubated fertile eggs of specific pathogen-free (SPF) chickens 20–23 h after fertilization. A total of 30 embryos were used across three independent experimental replicates (*n* = 3). This strategy was applied consistently for ESCs, MSCs, and satellite cells.

A piece of filter paper with a central aperture was placed gently onto the vitelline membranes, in order to frame the blastoderm. Afterwards, the vitelline membranes around the filter paper were cut, washed with Dulbecco’s phosphate-buffered saline (DPBS; Thermo Scientific) containing 1% antibiotic-antimycotic (Thermo Scientific) to remove the yolk. The cells were centrifuged and then filtered through 100, 70 e 40 μm strainer (Corning). The cells were collected by centrifugation and resuspended in Dulbecco’s modified Eagle’s medium (DMEM) low glucose (Thermo Scientific) supplemented with 10% fetal bovine serum (FBS; Thermo Scientific) and 1% antibiotic-antimycotic (Thermo Scientific).

### 2.2 Chicken mesenchymal stem cells and muscle satellite cells isolation

Embryonated SPF chicken eggs were incubated at 37.5°C with 55% relative humidity for 15 days and selected by candling. A total of 120 embryos were utilized in three independent experimental replicates (*n* = 3). Mesenchymal stem cells (MSCs) and satellite muscle cells were subsequently isolated from the thoracic and hind limb muscles of 15-embryonic-day chicken embryos. Muscles from the thorax and hind limbs were collected and washed with DPBS (Thermo Scientific) containing 1% antibiotic-antimycotic (Thermo Scientific). The muscle tissue was cut into small fragments using scissors on a glass plate. The minced tissue was dissociated using 0.1% collagenase type I (Thermo Scientific), incubated at 37°C for 1 h, and during digestion, gently triturated by suction in a syringe with an 18-gauge needle, 10 times every 15 min, and after that, centrifuged. Subsequently, the digestion tissue was incubated with 0.25% trypsin–EDTA (Thermo Scientific) at 37°C for 20 min, and then FBS (Thermo Scientific) was added to neutralize trypsin, and centrifuged. The cell suspension was filtered through 100, 70, and 40 μm strainer (Corning), and centrifuged. Afterward, the red blood cells were lysed using Pharm Lyse™ Buffer (BD Biosciences), incubated for 10 min at 4°C, added to DMEM medium, and centrifuged. The cells were cultured in a grown medium composed of DMEM high glucose medium (Thermo Scientific) supplemented with 20% FBS (Thermo Scientific) and 1% antibiotic-antimycotic (Thermo Scientific), at 37°C under 5% CO_2_. The cells were plated in T75 flasks, and after 2 h the adherent cells were obtained and identified as MSCs. The supernatant containing non-adherent cells was collected to proceed with the selective adhesion of chicken muscle satellite cells.

### 2.3 Stem cell characterization

To evaluate their multipotent characteristics, chicken MSCs were differentiated into adipogenic, chondrogenic, and osteogenic lineages utilizing standard induction media as per the manufacturer’s instructions (StemPro Adipogenesis, StemPro Chondrogenesis, StemPro Osteogenesis; Invitrogen). MSCs were seeded at a density of 1 × 10^4^ cells/cm^2^ in 10 cm^2^ plates and cultivated for 14 days for adipogenic and chondrogenic differentiation and for 21 days for osteogenic differentiation (data not shown). Cells were maintained at 37°C in a humidified atmosphere containing 5% CO_2_, with media changes performed bi-daily. Differentiation was evaluated with Oil-Red-O (Sigma-Aldrich), Alizarin-Red (Sigma-Aldrich), and Alcian Blue (Sigma-Aldrich) staining methods (19). Visual differences between differentiated and non-differentiated spheroids, as well as between spheroids and monolayers were examined using FIJI (version 2.14.0/1.54f). Additionally, stem cells were characterized by analyzing gene expression profiles, as detailed in the Quantitative RT-PCR section.

### 2.4 Chicken muscle satellite cell culture and cell differentiation

The muscle satellite cells were selected by selective adhesion. The collected supernatant was plated in new T75 flasks and cultured for 1 day. This supernatant was transferred to new T75 flasks and cultured for another day. The following day, adherent cells were detached with 0.05% trypsin–EDTA for 5 min, centrifuged and resuspended in fresh medium, plated in new T75 flasks, and cultured for 1 h. The cell suspensions were centrifuged and resuspended in a growth medium with 5 ng/mL recombinant human basic fibroblast growth factor (bFGF; Thermo Scientific). The muscle satellite cells were cultured at 37°C under 5% CO_2_ and sub-cultured when they reached 70% confluency. At this stage, the proliferating muscle satellite cells were considered committed myoblasts. For cell differentiation, when the myoblasts reached 90% confluence, they were cultured in a differentiation medium composed of DMEM high glucose medium (Thermo Scientific) supplemented with 2% FBS (Thermo Scientific) and 1% antibiotic-antimycotic (Thermo Scientific), at 41°C under 5% CO_2_, to induce the formation of myotubes and myofibers.

### 2.5 Differentiation of chicken mesenchymal stem cells to adipocyte-like cells

Chicken MSCs were seeded at 6 × 10^3^ cells/cm^2^ in T75 flasks and 24-well plates with DMEM high glucose medium (Thermo Scientific) supplemented with 10% FBS (Thermo Scientific) and 1% antibiotic-antimycotic (Thermo Scientific), at 39°C under 5% CO_2_. When the cells reached 60% confluence, the medium was changed to induce transdifferentiation. The transdifferentiation medium was composed of DMEM/F12 containing 12 μg/mL of soy lecithin (L-*α*-Phosphatidylcholine). After 7 days the medium was supplemented with 10 μg/mL of insulin (Invitrogen). The transdifferentiation medium was replaced every 2 days for 21 days.

### 2.6 Microscopy of lipid accumulation

The differentiated cells were fixed with 4% paraformaldehyde at pH 7.4 for 30 min and washed three times with DPBS on day 21. Lipid staining was performed using two methods: HCS LipidTOX Red Neutral Lipid Stain (Thermo Scientific), diluted 1:1000 according to the manufacturer’s protocol, and Nile Red staining, prepared by diluting the stock solution (1 mg/mL) to a working concentration of 0.5 μg/mL in DPBS. For both staining methods, the nuclei of the cells were counterstained with Hoechst 33342 (Thermo Scientific) at 1 μg/mL in DPBS for 10 min at room temperature. After staining, the cells were washed with DPBS and imaged under fluorescence light microscopy (EVOS M7000 Imaging System, Thermo Scientific).

### 2.7 Immunofluorescence staining and imaging

Chicken myoblasts and myotubes were fixed with 4% paraformaldehyde at pH 7.4 for 20 min at room temperature and washed three times with 0.1% Tween-20 in PBS. Fixed cells were permeabilized with 0.5% Triton X-100 for 15 min and washed three times with 0.1% Tween-20 in PBS. Cells were blocked with 5% goat serum or 3% bovine serum albumin (BSA) for 1 h at room temperature and washed three times with 0.1% Tween-20 in PBS. Subsequently, the primary antibodies were added separately (Supplementary Table S1): mouse monoclonal *anti-Pax7* conjugated Alexa fluor 488 (Santa Cruz Biotechnology) diluted 1:50, mouse monoclonal *anti-Myogenin* (F5D, Santa Cruz Biotechnology) diluted 1:50, mouse monoclonal *anti-Desmin* (D33, Thermo Scientific) diluted 1:50, mouse monoclonal *anti-MyoD* (5.8A, Thermo Scientific) diluted 1:100, mouse monoclonal *anti-Myosin 4* (MF20, Thermo Scientific) diluted 1:100, rabbit polyclonal *anti-Myf5* (Abcam) diluted 1:100, and rabbit polyclonal *anti-ITGA7* (Sunlong Biotech) diluted 1:100 in 1% BSA and 0.1% sodium azide, and incubated overnight at 4°C. Thereafter, the cells were washed three times with 0.1% Tween-20 in PBS, incubated with the secondary antibodies goat anti-mouse IgG or goat anti-rabbit IgG conjugated Alexa Fluor 488 (Thermo Scientific) diluted 1:800 in 1% BSA and 0.1% sodium azide, for 1 h at 37°C, and washed three times with 0.1% Tween-20 in PBS. F-actin was counterstained with Rhodamine Phalloidin (Thermo Scientific) diluted 1:500 in DPBS for 30 min at room temperature, and nuclei were counterstained with Hoechst 33342 (Thermo Scientific) diluted to 1 μg/mL in DPBS for 10 min at room temperature, and washed three times with 0.1% Tween-20 in DPBS. Stained cells were visualized under fluorescence light microscopy (EVOS M7000 Imaging System, Thermo Scientific).

### 2.8 Quantitative RT-PCR

For genetic characterization, total RNA from chicken cells was extracted using TRIzol (Invitrogen) associated with the RNeasy Mini Kit (Qiagen), according to the manufacturer’s recommendations. DNA digestion was performed on the column using RNase-Free DNase Set (Qiagen). RNA samples were quantified using the NanoDrop 2000 spectrophotometer (Thermo Scientific). Complementary DNA (cDNA) was synthesized using the SuperScript III First-Strand Synthesis SuperMix (Invitrogen), according to the manufacturer’s instructions. RT-qPCR reactions were performed using the QuantiNova SYBR Green PCR Kit (Qiagen), with concentration adjustments of each primer set. Gene expression analysis was performed using primers as specified in Supplementary Table S2.

In chicken embryonic stem cells (blastoderm), mesenchymal stem cells and myoblasts, the pluripotency genes were evaluated: chicken (c) *cOCT4*, *cSOX3*, *cNANOG*, *cSALL4* and *cCLDN3*, using primer sets described by Giotis et al. (20); and *cKIT* and *cLIN28A*, described by Han et al. (21). In dedifferentiated adipocytes, the genes *cPPARG*, *cADIPOQ*, *cPCK1*, *cADRP*, and *cFABP4* were evaluated, using primer sets described by Pasitka et al. (17). In chicken myoblasts and myotubes, the following genes were evaluated: *cPAX7* and *cMYOD*, using primer sets described by Hong and Do (22) *cMYMK* and *cMYH1E*, described by Ju et al. (23). In myoblasts and myotubes, the extracellular matrix genes were evaluated: *cCollagen I α1*, *cCollagen I α2*, *cLaminin*, *cFibronectin*, and *cElastin*, using primers sets described by Ma et al. (24).

Verification of chicken species DNA was performed by RT-qPCR for the *MT-CYB Gallus gallus* gene, described by Pasitka et al. (17), in primary chicken cells (dedifferentiated adipocytes, myoblasts, and myotubes).

The runs were executed on an ABI 7500 Real-Time PCR System (Applied Biosystems), and each sample was amplified in triplicate using 50 ng of cDNA. Relative gene expression was calculated using the formula 2^-∆Ct^ after normalization with the reference gene *cTBP*.

### 2.9 Nutritional analysis

Total protein content in chicken myoblasts was quantified via Dumas method, using Leco FP-528 (St. Joseph, Michigan, USA) equipment, following the AOAC Official Method 992.15. For protein determination, 0.2 g (± 0.0001) of cells were weighted in a tin (Sn) crucible, then placed in the autosampler carousel for further decomposition at 850°C at O_2_ atmosphere. Nitrogen content was determined by external calibration with an analytical calibration curve prepared with EDTA (Leco calibration sample P/N 502/092). The nitrogen content was subsequently converted to protein content using an appropriate nitrogen-to-protein conversion factor, ensuring accurate and reliable results.

### 2.10 Biomass production

To evaluate different strategies for cell biomass generation applicable to cultured meat, we designed a two-phase experimental approach. In the first phase, muscle and adipose biomasses were independently produced through monolayer cultures, followed by transglutaminase-induced tissue assembly. In the second phase, we explored three-dimensional (3D) co-culture formats by forming spheroids containing both differentiated myoblasts and adipocytes. These spheroids were subsequently seeded onto microcarriers to assess their potential for scalable 3D culture. The performance of microcarrier-based 3D co-cultures was compared with conventional 2D monolayer cultures in terms of viability, morphology, and cell integration.

To produce structured muscle and adipose tissue-like biomass, chicken myoblasts and MSCs were first differentiated separately in monolayer cultures. For muscle biomass, myoblasts were cultured in T75 flasks with high-glucose DMEM (Thermo Scientific) supplemented with 10% FBS (Thermo Scientific) until reaching 60% confluence. Myogenic differentiation was induced by reducing serum concentration to 2%, leading to the formation of aligned myotubes. These differentiated myofibers were then manually assembled and incubated overnight at 39°C with a 15% transglutaminase solution to promote crosslinking and generate a compact, tissue-like muscle biomass. For adipogenic biomass, MSC were cultured under adipogenic conditions using medium supplemented with 12 μg/mL soy lecithin and 10 μg/mL insulin. After the 14-day differentiation period, adipocytes were harvested, washed twice with DPBS (Thermo Scientific), and similarly treated with transglutaminase to produce adipose biomass.

After generating muscle and adipose biomass through monolayer culture without microcarriers, alternative strategies for three-dimensional (3D) cell biomass production were explored. Spheroids were generated composed both differentiated adipocytes and myoblasts (Supplementary Figure S2) were formed and subsequently co-cultured on commercial microcarriers (Cellva Ingredients, Brazil). These 3D cultures were then evaluated and compared to conventional two-dimensional monolayer cultures. Spheroid synthesis was performed by the liquid overlay method, which was introduced into each well of a 24-well plate (Corning) using micro-molds (MicroTissue® 3D Petri Dish®, 256 positions, Sigma-Aldrich). For this purpose, 1 × 10^6^ cells were seeded and incubated under humidified conditions with 5% CO_2_. Thus, the implanted cells spontaneously aggregated into three-dimensional spheroids, which were collected 2 days later. Spheroids and monolayer cultures were maintained in Roswell Park Memorial Institute (RPMI) 1,640 medium (Gibco), supplemented with 2% FBS, until subsequent processing.

To assess cell viability, the second-day aggregates were dissociated using 50 μL of TrypLE Select (Gibco, Thermo Fisher Scientific) and subsequently incubated for a period of 4 h at 37 C in an atmosphere containing 5% carbon dioxide. Cell viability was assessed using the Trypan Blue (Sigma-Aldrich) exclusion method. Cells were mixed in a 1:1 ratio with 0.4% Trypan Blue solution and counted using a hemocytometer. Viable (unstained) and non-viable (blue-stained) cells were quantified, and viability was calculated as the percentage of live cells relative to the total number of cells counted. After the incubation period, each aggregate was separated using a micropipette and examined under an optical microscope in order to verify complete disaggregation. To determine the rate of apoptotic cells, cells from spheroids were analyzed using the APO-DIRECT™ Kit (Invitrogen). Additionally, cells were stained with DAPI 1/2000 for 2 h and 30 min and LIVE/DEAD cell (Thermo Scientific) viability staining was used to assess live and dead cells, according to the product manual.

### 2.11 Chicken myoblasts cultivation on commercial microcarriers

To evaluate the adhesion of chicken myoblasts to commercial microcarriers as a preliminary step for potential cell culture scale-up, we selected microcarriers provided by Cellva Ingredients.

Microcarriers were prepared according to the manufacturer’s instructions. Initially, the storage solution was removed, and the microcarriers were washed in DPBS (Thermo Scientific). This washing step was repeated to ensure thorough cleaning. The DPBS (Thermo Scientific) was removed, and the microcarriers were transferred to a sterile plate. Subsequently, the microcarriers were equilibrated in the culture medium by adding 1 mL of medium per gram of microcarriers.

Chicken myoblasts were previously cultured in T75 flasks using DMEM high glucose (Thermo Scientific) supplemented with 10% FBS (Thermo Scientific), 100 U/mL penicillin, and streptomycin (Thermo Scientific), under a humidified atmosphere of 5% CO_2_ at 39°C. Upon reaching confluency, the cells were detached using 0.25% trypsin–EDTA (Thermo Scientific), resuspended in a medium, and then used for the experiments.

The myoblasts were then seeded onto the microcarriers at a density of 3 × 10^6^ cells per gram of microcarriers. The cell suspension was incubated with the microcarriers in a small volume of medium for at least 3 h to allow for initial cell adhesion to the material. After this period, an additional medium was added, and the culture was maintained for 4 days, with medium changes every 2 days. The morphology of the myoblasts on the microcarriers was observed under light microscopy (EVOS M7000 Imaging System, Thermo Scientific).

### 2.12 Statistical analysis

Gene expression levels obtained from RT-qPCR were analyzed using the 2^-ΔCt^ method with normalization with the reference gene cTBP. Data obtained from the experimental procedures were analyzed using GraphPad Prism 10.5. For two groups comparison, differential expression analysis was performed using a Welch’s t-test, while for three groups comparison, a Kruskal-Wallis followed by Dunn’s correction was used. A *p*-value ≤ 0.05 was considered as significant.

## 3 Results

### 3.1 Chicken mesenchymal stem cells and embryonic stem cells proliferation

The culture of chicken stem cells was stable, with the ability to self-renew and differentiate into different cell types, such as the transdifferentiation into adipocytes demonstrated in this study. The embryonic stem cells were maintained in feeder-free conditions and exhibited a doubling time of 18–24 h between passages 3 and 8. In contrast, mesenchymal stem cells derived from embryonated eggs maintained a consistent doubling time of approximately 26–28 h during the same passage range under standard culture conditions.

In the genetic characterization of primary cells isolated from chicken embryos, the expression of pluripotency genes varied among the different types of chicken cells. Embryonic stem cells (blastoderm), which represent an early stage of embryonic development, exhibited high expression of the evaluated genes, reflecting their pluripotency. In contrast, mesenchymal stem cells, which possess the capacity for self-renewal and differentiation, showed moderate expression, demonstrating their restricted pluripotency.

### 3.2 Myogenic differentiation of muscle satellite cells into myotubes

**Figure 1:**
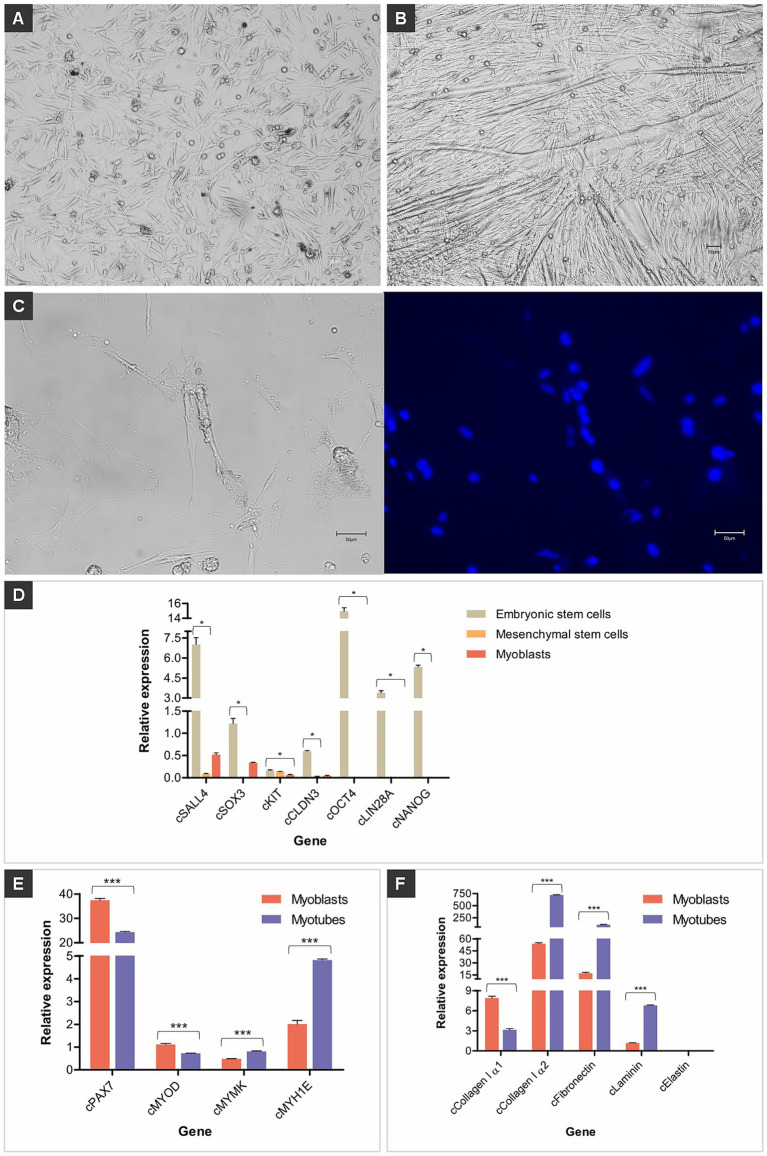
Chicken muscle cells. **(A)** Representative images of established chicken myoblasts derived from muscle tissues; scale bar 50 μm. **(B)** Representative images of established chicken myotubes obtained by myoblast differentiation; scale bar 50 μm. **(C)** Long tubular and multinucleated chicken myotube in bright field and counterstained with Hoechst 33342 (blue); scale bar 50 μm. **(D)** Relative gene expression of pluripotency genes (*cSALL4*, *cSOX3*, *cKIT*, *cCLDN3, cOCT4*, *cLIN28A*, and *cNANOG*), **(E)** muscle genes (*cPAX7*, *cMYOD*, *cMYMK*, and *cMYH1E*), and **(F)** extracellular matrix genes (*cCollagen I α1*, *cCollagen I α2*, *cFibronectin*, *cLaminin*, and *cElastin*) in primary cells isolated from chicken embryos. Data in **(D–F)** are shown as mean plus standard deviation. One asterisk indicates *p* ≤ 0.05, two asterisks indicate *p* ≤ 0.01 and three asterisks indicate *p* ≤ 0.001.

Chicken myoblasts were derived from the primary culture of chicken embryo muscle tissue. The established myoblasts presented typical myoblast morphology, which is a fibroblast-like shape with a slightly smaller size (Figure 1A). The cells elongated after reaching confluence and being cultured in a differentiation medium. Cell elongation is a sign of myogenesis, which is the result of fusion between myoblasts, forming linear and multinucleated myotubes (Figures 1B,C). To confirm that these established cells were myoblasts and myotubes, they were further characterized by gene expression and phenotypic analysis. Myoblasts, muscle precursor cells committed to differentiating into muscle fibers, exhibited low or undetectable expression of low or undetectable expression of pluripotency markers, indicating a loss of pluripotency and functional specialization (Figure 1D).

The expression of the evaluated genes in chicken myoblasts and myotubes regulates the processes of proliferation, differentiation, and cell fusion that form muscle tissue. The *cPAX7* and *cMYOD* genes were more highly expressed in myoblasts, and the expression of these genes decreased in myotubes (Figure 1E). The *cMYMK* and *cMYH1E* genes had low expression in myoblasts, and their expression increased in myotubes (Figure 1E).

The extracellular matrix (ECM) is essential for the development, organization, and functionality of these cells. Chicken myoblasts and myotubes play a central role, as they are the precursors of muscle fibers. The genes *cCollagen I α1*, *cCollagen I α2*, *cLaminin* and *cFibronectin* were highly expressed in myoblasts and myotubes, while the Elastin gene was lowly expressed (Figure 1F). RT-qPCR for the mitochondrially encoded cytochrome B (*MT-CYB*) gene showed that the primary cells isolated and differentiated here originate from the species *Gallus gallus* (data not shown). Culturing the myoblasts in differentiation medium activated transcription factors, promoting the fusion of several precursor cells to form myotubes, which subsequently developed into myofibers (Supplementary Video 1).

**Figure 2:**
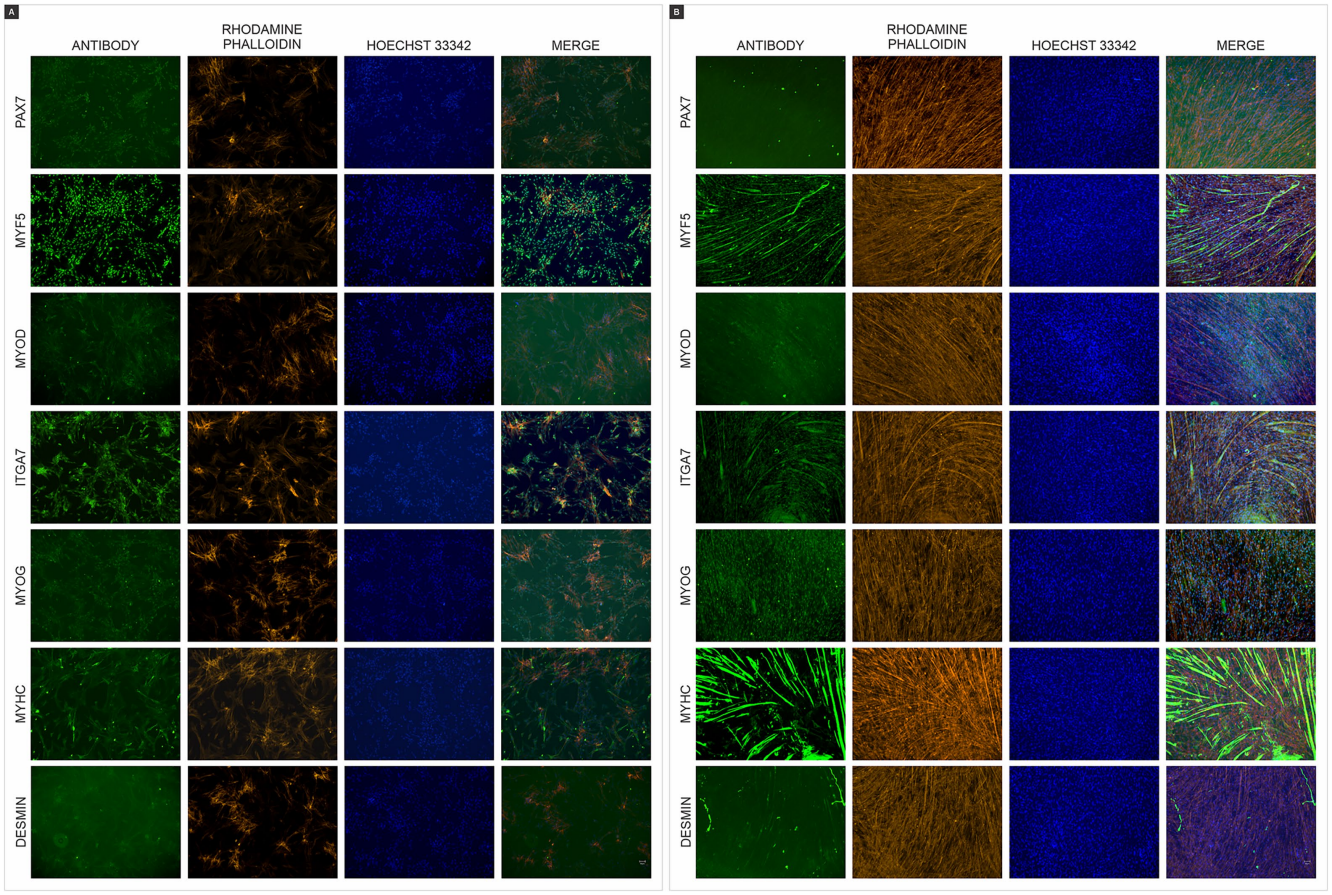
Immunofluorescence of chicken muscle cells. **(A)** Immunofluorescence staining of chicken myoblasts **(B)** and chicken myotubes with antibodies (green) paired box 7 (*PAX7*), myogenic factor 5 (*MYF5*), myogenic determination (*MYOD*), integrin alpha 7 (*ITGA7*), myogenin (*MYOG*), myosin heavy chain (*MYHC*), and desmin (*DES*). F-actin was counterstained with Rhodamine Phalloidin (orange), and nuclei were counterstained with Hoechst 33342 (blue). Scales bar 50 μm.

Phenotypic analysis was performed using immunocytochemistry with immunocytochemical analysis using antibodies against paired box 7 (*PAX7*), myogenic factor 5 (*MYF5*), myogenic determination (*MYOD*), integrin alpha 7 (*ITGA7*), myogenin (*MYOG*), myosin heavy chain (*MYHC*), and desmin (*DES*), both in chicken myoblasts (Figure 2A) and chicken myotubes (Figure 2B). The myotubes exhibited more positive staining for *MYOG*, *MYHC*, and *DESMIN*, demonstrating that the myoblasts differentiated into linear and multinucleated myotubes.

### 3.3 Chicken mesenchymal stem cells differentiation into adipocytes

**Figure 3:**
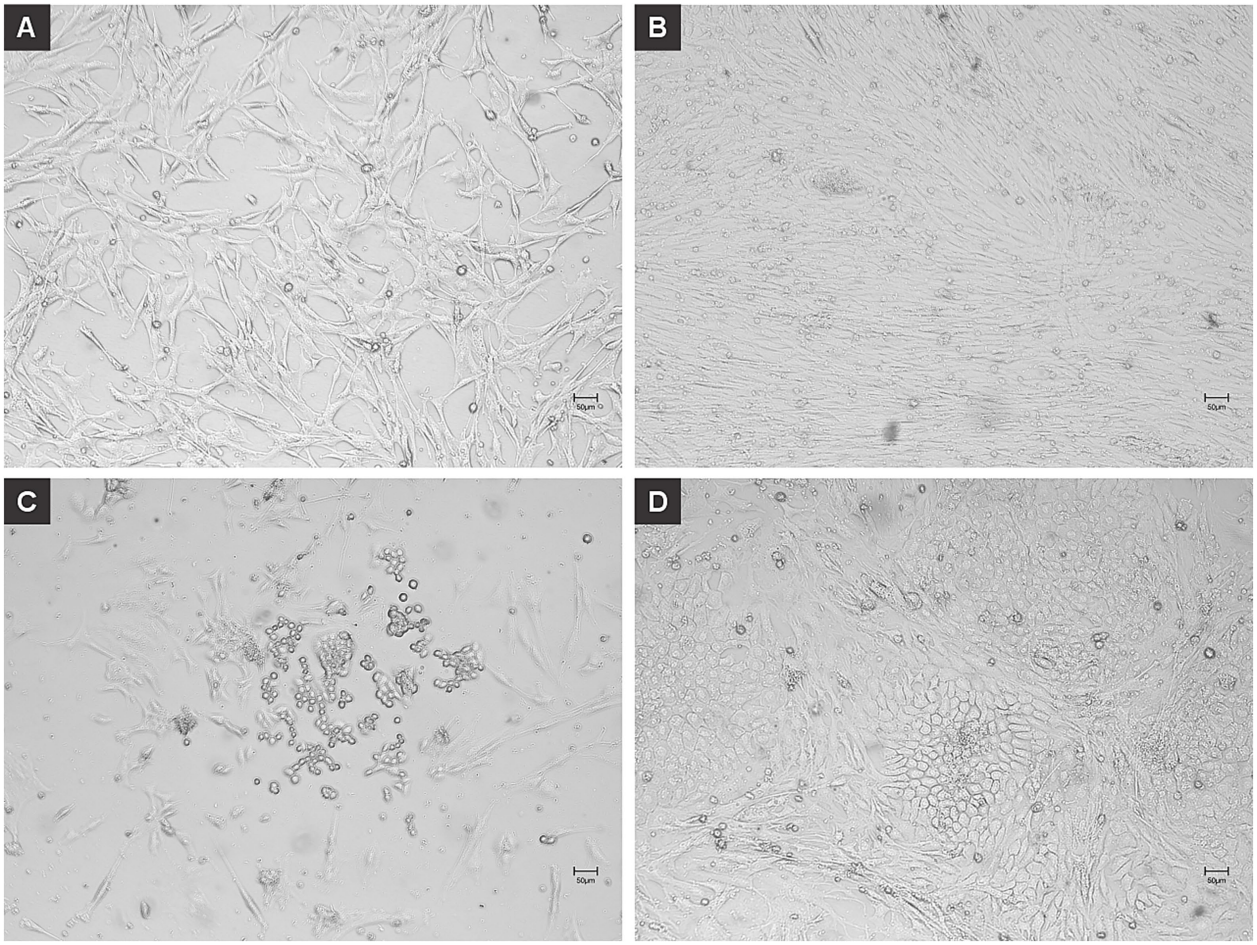
Adipogenic dedifferentiation of chicken mesenchymal stem cells (MSCs). Chicken MSCs were induced to dedifferentiate into adipocytes using L-α-Phosphatidylcholin and monitored over time. **(A)** At 0 h (day 0), undifferentiated MSCs displayed a fibroblast-like, spindle-shaped morphology typical of early-passage mesenchymal cells. **(B)** By day 4, the cells exhibited increased confluency and subtle morphological changes, with some adopting a more rounded shape indicative of early adipogenic commitment. At this early stage of induction, the cells are still proliferating and beginning to undergo morphological changes. The culture appears confluent as the MSCs maintain their fibroblast-like morphology and high proliferative capacity. **(C)** On day 6, a greater number of cells showed a rounded morphology along with the initial formation of intracellular vesicles, characteristic of early adipocytes. At this intermediate differentiation stage, some cells begin to round up and accumulate lipid droplets, a hallmark of early adipogenic commitment. During this transition, many cells detach or die, possibly due to their sensitivity to the induction medium or mechanical stress from media changes. This explains the apparent reduction in cell density compared to Panel B. Additionally, some lipid-filled cells may not adhere strongly to the surface, contributing to reduced confluency. **(D)** By day 11, cells had adopted a mature adipocyte-like phenotype, with prominent intracellular lipid droplets and a spherical shape. In the later stage of differentiation, the remaining adherent cells have adapted to the adipogenic conditions and completed differentiation. They exhibit robust lipid accumulation and a mature adipocyte-like phenotype. These cells tend to reoccupy the culture surface, and this contributes to the nearly confluent appearance seen here. Scales bar 50 μm.

**Figure 4:**
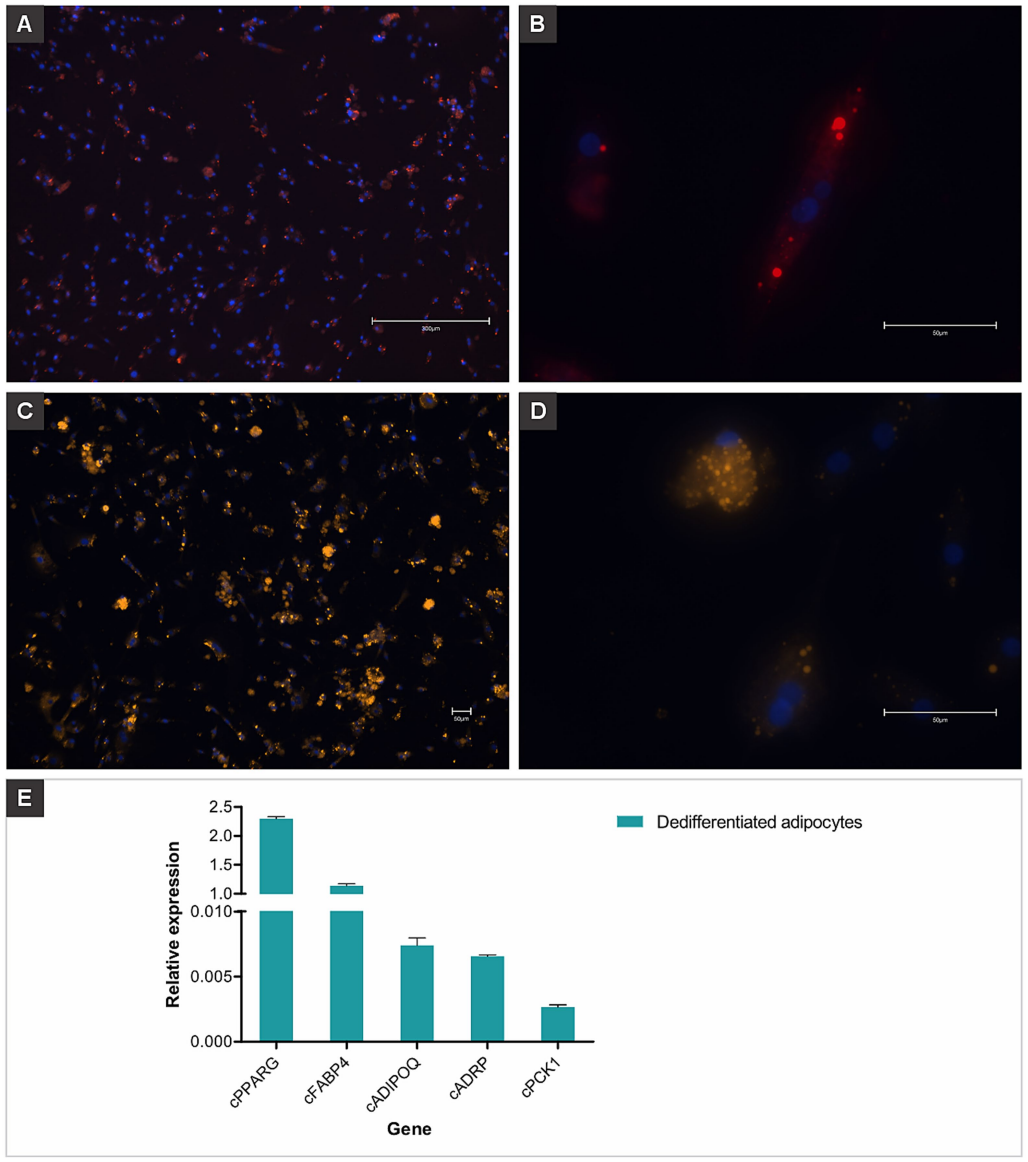
Dedifferentiated chicken adipocytes derived from mesenchymal stem cells. Fluorescent staining was used to visualize lipid accumulation and confirm adipogenic differentiation. **(A)** Dedifferentiated adipocytes stained with HCS LipidTOX™ Red Neutral Lipid Stain, showing widespread lipid droplet distribution throughout the culture. Nuclei were counterstained with Hoechst 33342 (blue). Scale bar: 300 μm. **(B)** Higher magnification of a single cell showing intracellular lipid droplets stained in red (LipidTOX) and nucleus in blue (Hoechst). Scale bar: 50 μm. **(C,D)** Cells stained with Nile Red, highlighting numerous intracellular lipid droplets as bright orange/yellow signals. Nuclei were counterstained with Hoechst 33342 (blue). Scale bars: 50 μm. **(E)** Relative gene expression of adipogenic markers (*cPPARG*, *cFABP4*, *cADIPOQ*, *cADRP*, and *cPCK1*) in dedifferentiated chicken adipocytes, confirming the adipogenic phenotype at the molecular level. Data are shown as mean plus standard deviation.

Within 4 days of adipogenic induction, chicken MSCs adopted the characteristic rounded morphology of adipocytes (Figure 3). On day 14, lipid staining using HCS LipidTOX Red Neutral Lipid Stain (Figures 4A,B) and Nile Red (Figures 4C,D) validated lipid accumulation. RT-qPCR analysis showed pronounced transcriptional up-regulation of *cPPARG*, while *cFABP4*, *cADIPOQ* and *cPCK1* were also expressed at elevated levels (Figure 4E).

### 3.4 Chicken muscle and adipogenic biomass

**Figure 5:**
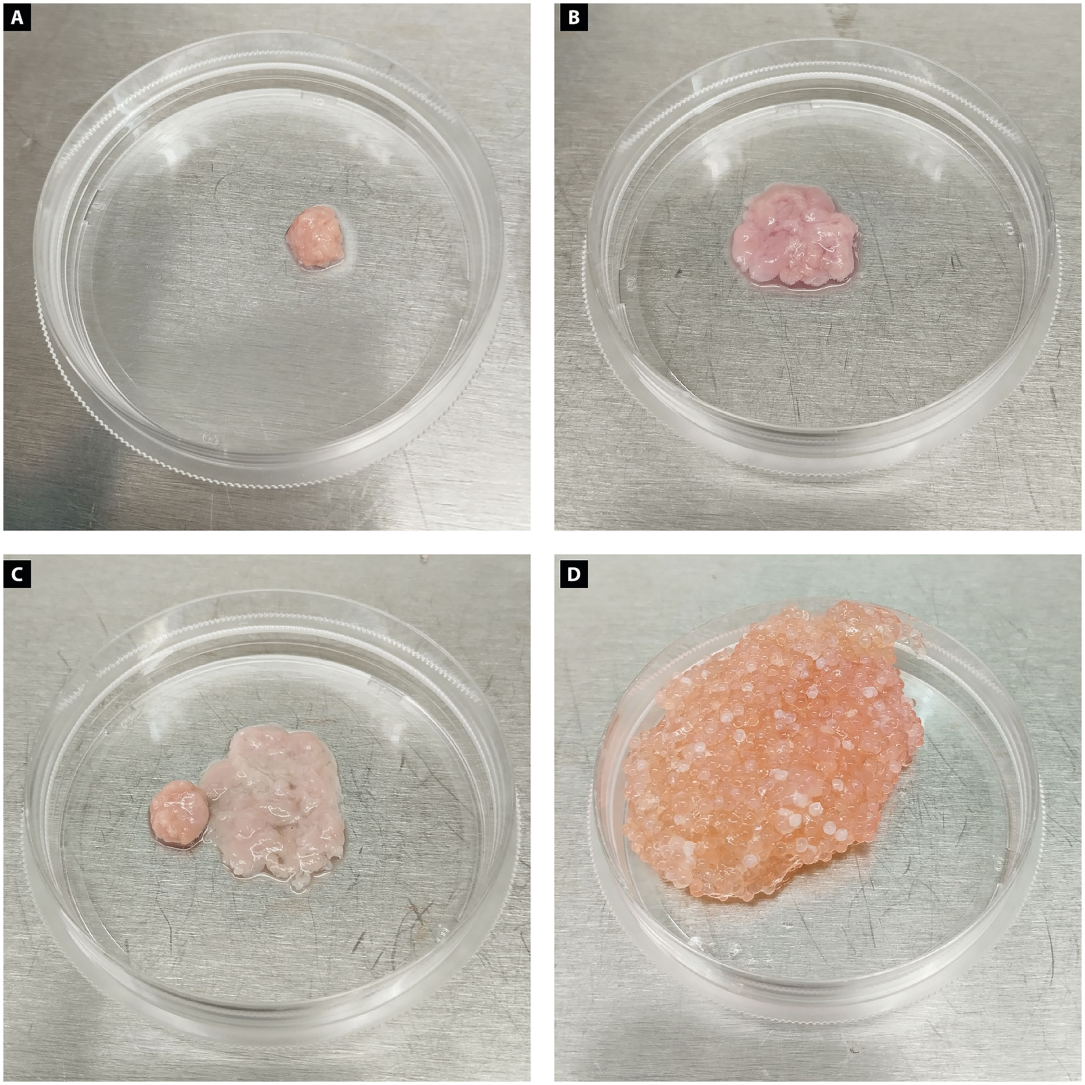
Chicken biomass constructs produced from muscle and adipose cells. **(A)** Muscle biomass produced from differentiated chicken myoblast appears compact, with a dense and uniform structure and a pink coloration typical of muscle tissue. **(B)** Adipogenic biomass generated from dedifferentiated chicken adipocytes presents a looser, more irregular morphology with a paler, translucent appearance, consistent with lipid-rich tissue. **(C)** Chicken muscle and adipose biomasses incorporating microcarriers are shown side by side, highlighting their morphological differences in structure, size, and consistency. **(D)** Combined muscle and adipose chicken biomass integrated with microcarriers, resulting in a larger, heterogeneous construct with a granular texture. The structure displays a mixture of pink and orange tones, reflecting the presence of both muscle and fat components, as well as the incorporated microcarriers that contribute to the bulk and support of the tissue.

The construct demonstrated structural integrity and scalability. Approximately 0.3 g of muscle biomass was obtained after culturing chicken myoblasts for 30 days, followed by mechanical harvest and incubation using 15% transglutaminase solution. Similarly, adipogenic biomass, derived from MSCs differentiated into adipocytes, yielded approximately 0.5 g after 14 days of adipogenic induction. Both biomass types were processed into macroscale constructs, demonstrating structural integrity and scalability (Figure 5).

The spheroids were spherical in shape and ranged from 200 to 300 μm in diameter 24 h after the start of their production. The area of the spheroids prepared from 40,000 cells was 0.306 ± 0.022 mm^2^ for proper nutrient diffusion, which resulted in cell viability of 94.05%. Additionally, spheroids did not affect the cell viability in terms of apoptosis since ranged above 90%, except for the positive control (<30%). No necrotic centers were detected in the spheroids. The spheroids had discrete areas of necrosis or apoptosis in the proliferative zone and an increased density of dead cells in the quiescent zone, aligning with the typical biology of the spheroids.

**Figure 6:**
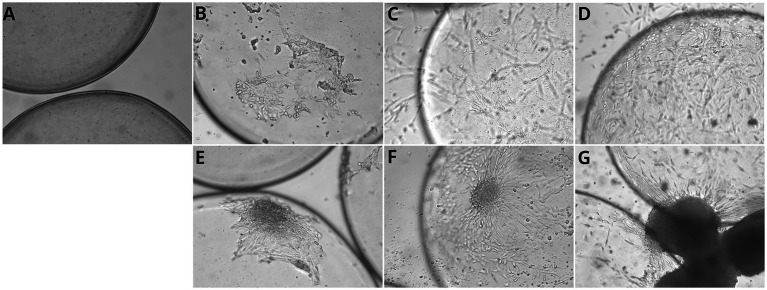
Myoblast proliferation on commercial microcarriers over time in monolayer and spheroid cultures. **(A)** Cellva Ingredients (Brazil) microcarriers incubated without cells, showing the baseline structure and surface morphology. **(B–D)** Myoblasts cultured in monolayer on microcarriers. **(B)** After 24 hours of incubation, initial attachment of chicken myoblasts is observed on the microcarrier surface. **(C)** At 24 hours, a noticeable increase in cell coverage occurs, with myoblasts beginning to spread and form early connections. **(D)** By 96 hours, microcarriers are densely colonized by proliferating myoblasts, exhibiting extensive cell spreading and aggregation, indicating robust attachment and expansion. **(E–G)** Myoblasts cultured as spheroids on microcarriers. **(E)** At 1 hour, initial attachment of spheroid-associated cells begins. **(F)** By 24 hours, partial spreading and integration of spheroids with the microcarrier surface is visible. **(G)** At 96 hours, microcarriers exhibit large spheroid clusters, demonstrating strong aggregation and proliferation. Scale bar = 50 μm.

The spheroids adhered to the microcarriers within 24 h (Figures 6E–G). Notably, following adhesion, the spheroid-derived cells spread and colonized the microcarriers more rapidly than cells cultured under conventional monolayer conditions (48 h versus 80 h).

### 3.5 Nutritional analysis of myoblast protein content

Analysis revealed that the cultured myoblasts contained 10.63% ± 0.29 total protein on a dry weight basis.

### 3.6 Isolated chicken myoblasts adhesion to microcarriers

After 24 h of incubation, primary chicken myoblasts were observed to adhere efficiently to microcarriers. By 96 h, an increased density of adhered cells was evident, indicating not only sustained adhesion but also active proliferation over time. These observations suggest that the evaluated microcarriers provide a suitable surface for myoblast attachment and expansion, supporting their potential use in large-scale bioprocesses for cultivated meat production (Figures 6B–D).

## 4 Discussion

To create mimetics of conventional meat using cell culture for protein production from animal cells, the primary goal is to develop mature muscle tissue. Muscle tissue is composed of myofibers (myotubes), long, multinucleated cells that contract to generate force and enable movement. Besides myofibers, muscle tissue contains other essential cell types, including fibroblasts, adipocytes, and blood vessels, all of which contribute significantly to the structural integrity, function, and nutrients and oxygen delivery of the tissue, all of which contribute significantly to the structural integrity and function of the tissue. Within this complex cellular microenvironment, ECM proteins play a critical role in providing structural support, mediating cell adhesion and communication, and preserving the architectural integrity of the tissue (25). Together, these components give skeletal muscle tissue its distinct properties of skeletal muscle tissue in meat. However, it is still challenging to recreate these characteristics *in vitro*, as in the production of cultured meat. A primary difficulty lies in co-culturing diverse cell types, each with unique characteristics and requirements. In this study, we explored a culture approach by deriving multiple meat components—muscle, and fat—from two cell types and characterizing the resulting tissues. Specifically, we utilized chicken embryonic and mesenchymal stem cells to generate muscle tissue, and fat storage in a carefully regulated monolayer culture environment, as well as in three-dimensional cultures using microcarriers. This approach allowed us to cultivate meat with the desired characteristics, proliferating both embryonic and mesenchymal stem cells in the desired quantity and then differentiating them, thus advancing the development of cultured meat with greater similarity to its conventional counterpart.

The ageing of meat, that is, the transformation of muscle into meat, represents a major limitation for cultivated meat production, mainly because the critical post-mortem metabolic and biochemical processes that occur naturally in conventional meat are either poorly understood or absent in cultured muscle cells. Purslow (26) highlights that cultivated meat production generally involves harvesting cultured muscle cells that differ structurally and biochemically from whole muscle tissue because they lack connective tissue and the natural complexity of muscle fiber types. Cultured cells are often monocultures of embryonic muscle cells rather than adult muscle fibers, which differ in protein isoforms important for meat texture. In conventional meat, post-mortem metabolism after animal slaughter triggers complex biochemical changes such as pH decline, rigor mortis, proteolysis by enzymes (calpains and caspases), and changes in muscle protein structures. These transformations affect important sensory qualities like tenderness, flavor, juiciness, and color. The variability in these processes contributes significantly to meat quality and consumer acceptance.

The analysis of pluripotency gene expression revealed high expression levels in stem cells, pluripotent nature, and their ability to self-renew and differentiate into various cell types. Jean et al. (27) also demonstrated that embryonic cells expressed classical pluripotency-related genes, such as *OCT4*, *NANOG*, *SOX3*, and *SALL4*. Genes such as *OCT4* and *NANOG* play essential roles in maintaining the pluripotent state in chicken ESCs (28), ensuring their ability to remain undifferentiated. MSCs showed moderate expression compared to ESCs, reflecting more restricted pluripotency. MSCs are multipotent stem cells that have the ability to differentiate into osteocytes, chondrocytes, adipocytes, and myocytes (29). Here, MSCs were transdifferentiated into adipocytes as well as into muscle satellite cells, in which myoblasts differentiated into myotubes. Furthermore, the use of embryonic cells represented a critical step in establishing a robust and well-characterized primary cell model. As detailed in our study, these cells were cultured without feeder layers, particularly mouse fibroblasts, which are frequently used in other protocols and may raise regulatory concerns. By avoiding xenogeneic support systems and using avian-specific, traceable sources under controlled sanitary conditions, we aimed to minimize potential regulatory barriers.

The use of primary chicken embryonic cells and mesenchymal stem cells (MSCs) was strategic because they closely resemble the native physiological state and avoid the genetic or epigenetic alterations often seen in immortalized or reprogrammed cell lines (30). These primary cells exhibited favorable growth kinetics and high differentiation potential without genetic modification, allowing efficient commitment to the adipogenic and myogenic lineages. The cultured meat market requires well-characterized, immortalized chicken fat or muscle cell lines to achieve reproducible bioprocesses. Therefore, establishing well-characterized primary cell lines is a necessary step for future immortalization strategies, also considering the regulatory implications, since methods involving exogenous genes can classify the product as genetically modified. Similarly, iPSCs, although highly versatile, face regulatory and stability challenges due to their generation methods and long-term genetic instability (31). Therefore, multipotent primary cells were chosen for their robust proliferation, low senescence, and suitability to generate functional biomass for cultured meat.

Skeletal muscle tissue consists not only of mature, multinucleated muscle fibers but also a diverse array of supporting cell types. Muscle satellite cells, as myogenic progenitors, exhibit a robust regenerative capacity, differentiating readily into myotubes and mature myofibers. Environmental factors, particularly temperature, significantly influence the activity of muscle satellite cells, with effects that can either enhance or inhibit their functionality. In chickens, muscle satellite cell proliferation and differentiation are highly sensitive to changes in temperature (32, 33). In this study, a temperature of 41°C was applied to support satellite cell differentiation as well as myotube proliferation.

During the differentiation of muscle satellite cells into myofiber, we previously observed a large population of myoblasts cells, characterized by lower or absent expression of these pluripotency genes, reflecting their specialized role in muscle tissue formation. The progression of myoblasts into myofibers is marked by diminished pluripotency and increased expression of muscle-specific genes, underscoring their commitment to a muscle lineage. Our findings demonstrate that *cPAX7* and *cMYOD* genes are highly expressed in myoblasts, but their expression decreased substantially during differentiation, indicating a transition to a more differentiated phenotype, corroborating previous findings. The reduction in *PAX7* expression signals that progenitor cells have exited the proliferative state and are moving toward differentiation (34). This is consistent with the role of *MYOD* as a key regulator in the cell cycle transition to myogenic commitment, promoting the expression of differentiation-related genes (35). In contrast, the *cMYMK* and *cMYH1E* genes exhibit low expression in myoblasts but increase during differentiation into myotubes, underscoring their importance in cell fusion and muscle fiber maturation. *MYH1E*, an essential structural protein in mature muscle fibers, is widely used as a marker of differentiation. Similarly, *MYMK* is essential for the cell fusion process, facilitating the formation of multinucleated myotubes (36). Identification of cells belonging to the *Gallus gallus* species using RT-qPCR for the *MT-CYB* gene confirms the origin of the cells, ensuring the authenticity of the cultures. These results provide valuable insights into the molecular mechanisms underlying myogenesis and highlight the dynamic expression profiles of myogenic markers during muscle development.

The ECM plays a crucial role in the development, maintenance, and functionality of muscle cells, including both myoblasts and myotubes. It provides structural support, facilitates cell signaling, and organizes tissue architecture. The genes *cCollagen I α1*, *cCollagen I α2*, *cLaminin* and *cFibronectin* are essential for cell maintenance and differentiation. However, the low expression of *cElastin* reflects the specific microenvironment requirements of muscle tissue (37).

The screening of myoblast and myotube populations resulted in a culture of mononuclear cells with distinct morphology and protein expression patterns compared to muscle satellite cells (38). A robust immunocytochemistry approach was applied to characterize chicken myoblasts and myotubes, using key markers such as *PAX7, MYF5, MYOD, ITGA7, MYOG, MYHC*, and *DES*. These markers are essential in different stages of myogenesis, including proliferation, differentiation, and muscle fiber formation. The myotubes obtained after myogenic differentiation showed strong staining of *MYHC*, a terminal differentiation marker of skeletal muscle cells. Differentiation into sufficiently mature myofibers created through the fusion of myoblast cells, together with cell proliferation, are important parameters for the production yield and quality of cultured meat. In addition, myoblast-derived biomass showed nutritional viability, with a protein content of 10.63%, supporting its potential as a functional component for cultured meat formulations.

A recent metabolomic study revealed that the impact of amino acid metabolism suggests the nutritional composition of cultured meat may differ from conventional meat, indicating the need for future optimization (7, 17, 39, 40). Currently, most studies report that cultured chicken exhibits a lower protein content (14.8–18%) compared to conventional chicken (7, 40) which presents approximately 22.5% protein according to USDA data (2023). However, any cell-cultured meat product has the potential for controlled and adjustable protein content, including the possible incorporation of plant-based components to enhance bioavailability and nutritional value. For instance, Pasitka et al. (7) achieved cultured chicken with 22.6% protein by incorporating soy into the product. Moreover, cellular cultivation enables technological manipulation and personalization of other nutrients, such as lipids, to achieve the desired nutritional profile. Nevertheless, such manipulation was beyond the scope of the present study.

Beyond muscle protein, lipid content is a critical factor in the quality of meat. While muscle cells have a limited capacity to store fat, adipocytes are responsible for generating intramuscular fat, which constitutes approximately 80% of the fat in meat. This fat is critical for imparting juiciness, tenderness, and aroma to meat, with higher fat content enhancing the flavor during cooking (24, 41). Consequently, fat is primarily composed of adipocytes with a high concentration of lipid droplets, which are primarily deposited in fat cells within the tissue (24). To accurately replicate the sensory characteristics of intramuscular fat in cultured meat, co-culturing muscle and fat cells is essential. For instance, co-culturing preadipocytes with myoblasts can potentially elevate intramuscular fat content, improve tenderness, and enhance flavor intensity in the final product (42). However, co-culturing diverse cell types presents technical challenges, as each cell type requires a distinct, optimized environment to develop and differentiate effectively. Shared culture conditions may be suboptimal for one cell type, leading to hindered cell growth and efficiency (43). Research has shown that adipocytes developing in close proximity to muscle cells can modulate myogenesis, thereby influencing the development and characteristics of muscle tissue (44). To overcome these challenges, identifying the most suitable cell source and optimizing conditions for differentiation into either muscle or fat cells are critical steps in achieving the desired genotypic and phenotypic outcomes for cultured meat.

The adipogenic capacity of preadipocytes can be evaluated through alterations in transcription factor expression and cell cycle characteristics. The initially adhered MSCs were induced to differentiate into preadipocytes. Adipogenesis depends on the essential transcription factor peroxisome proliferator activating receptor gamma (*PPAR-γ*) (45). To turn on the adipogenic transcriptional program, *PPAR-γ* interacts with C/EBP family transcription factors (46). Together with lipogenic genes including fatty acid synthase (*FAS*) and fatty acid binding protein 4 (*FABP4*), mature adipocytes preserve the expression of *PPAR-γ*, widely regarded as an adipogenic marker. In differentiated adipocytes derived from chicken MSCs, the evaluated genes are associated with the formation and functionality of adipose tissue, which is essential for reproducing the sensory characteristics of meat. Gene expression analysis by RT-qPCR shows that *cPPARG* exhibited the highest expression, reflecting its critical role as a master regulator of adipogenesis (45). Genes such as *cFABP4* and *cADIPOQ*, associated with fatty acid transport and metabolic regulation, were expressed, indicating the acquisition of functional adipocyte properties (47). The expression of *cPCK1*, involved in lipid and glucose metabolism, validated the successful differentiation process. Based on these characteristics, we hypothesized that these cells may be fibro-adipogenic progenitor cells (*FAPs*) and confirmed their expression of *FABP4*, as well as other factors previously implicated in adipogenesis, including *PPAR-γ* and *ADIPOQ*. These *FAPs* demonstrated lipid droplet accumulation, as well as strong induction of adipocyte marker genes when treated with a differentiation medium containing adipogenic inducers. As *FAPs* are a primary source of intramuscular fat depots *in vivo*, we suggest that MSC-derived cultured fat could more accurately resemble traditional adipose tissue compared to fat produced from other cell types, such as fibroblasts. Notably, the lipid accumulation rate in MSC-derived *FAPs* was higher than those from broiler ESCs. Furthermore, *FAPs* can be efficiently co-cultured with muscle satellite cells, enhancing the potential for a viable bioprocess in cultured meat production. To further optimize cell sorting strategies, we conducted an extensive characterization of the immunogenotypic and phenotypic profiles of these *FAPs* cells.

Our findings demonstrate the feasibility of protocols for differentiating MSCs into adipocytes and producing cellular biomass with desirable characteristics for cultivated meat production. Adipogenic differentiation was validated through gene expression analysis and lipid accumulation, consistent with previous studies emphasizing the roles of genes such as *PPARG* and *FABP4* in adipogenesis and intracellular fatty acid transport (45, 46). Staining using HCS LipidTOX Red Neutral Lipid Stain and Nile Red revealed the presence of intracellular neutral lipids, which could be triglycerides and cholesterol esters, consistent with the maturation of adipocytes (48). Additionally, our use of lecithin as an adipogenic inducer is supported by the study of Pasitka et al. (17), who demonstrated that phosphatidylcholine, a key component of soy lecithin, activates *PPAR-γ* in chicken fibroblasts, effectively promoting adipocyte formation. Soy lecithin, rich in phosphatidylcholine (L-*α*-PC), was used as a food-grade adipogenic inducer previously shown by Pasitka et al. to activate PPARγ in chicken fibroblasts and promote lipid accumulation without insulin or dexamethasone. In our study, 12 μg/mL lecithin induced adipocyte-like morphology in chicken MSCs from day 4, with RT-qPCR confirming upregulation of *cPPARG*, *cADIPOQ*, *cFABP4*, and *cPCK1*. These results validate lecithin as a safe, effective inducer of adipogenesis for cultured meat applications. This approach eliminates the need for chemically restrictive inducers or hormones, such as insulin and dexamethasone, making it more suitable for food-grade applications.

The successful adhesion and proliferation of primary chicken myoblasts on microcarriers observed in this study demonstrate their potential as a platform for scalable muscle cell cultivation (47). After 24 h of incubation, cells were visibly adhered to the microcarriers, and a substantial increase in cell density was observed at 96 h, indicating active proliferation. These findings highlight the suitability of the tested microcarriers for dynamic suspension culture systems, such as stirred-tank bioreactors, which are essential for large-scale production of cultured meat (49). Microcarriers offer a significantly increased surface-to-volume ratio, allowing for higher cell yields in reduced volumes and enhanced process control compared to traditional planar systems (49). The observed compatibility between the chicken myoblasts and microcarrier surface suggests that microcarriers possess adequate surface chemistry, charge, and topography to support anchorage-dependent cell attachment, spreading, and expansion—critical for preserving cell phenotype and myogenic potential (49–51). Future studies should investigate the performance of these microcarriers under dynamic culture conditions, particularly focusing on their resistance to shear stress, capacity for bead-to-bead transfer, and detachment efficiency—parameters that are decisive for process scalability and downstream cell recovery (49, 52).

In addition to microcarrier-based expansion, transglutaminase-mediated consolidation of cellular biomass enhances the formation of 3D tissue-like constructs suitable for food applications. This method, which ensures the structural and sensory integrity of the biomass, is consistent with findings by Yuen et al. (53), who used transglutaminase to consolidate cultivated adipogenic cells, resulting in tissues with mechanical properties comparable to native adipose tissue. In our study, the combined use of mesenchymal stem cell transdifferentiation and transglutaminase stabilization enabled the production of lipid-rich biomass, akin to previous experiments, but with enhanced scalability potential for industrial applications. An additional noteworthy finding was the use of spheroids in microcarrier-based culture. Compared to monolayer cells, spheroids exhibited a higher proliferation rate and more efficient microcarrier colonization. Moreover, by the fifth day of culture, we observed the aggregation of microcarriers mediated by spheroid adhesion, which appeared to facilitate biomass formation. Nevertheless, it is important to note that blood vessels are an integral part of native muscle, and their absence in current *in vitro* systems remains a limitation for replicating thicker and more functional tissue.

In summary, we present a primary cell culture strategy to construct multicomponent tissues by developing myogenic and adipogenic microtissues derived from multipotent cells. For cultured meat production, the use of multipotent cells is particularly advantageous due to their higher proliferation rates, which enhance scalability and efficiency. Alternatively, the immortalization of primary cells, such as myoblasts and preadipocytes, represents another promising approach to ensure long-term cell availability and consistent performance in cultured meat applications.

## Data Availability

The original contributions presented in the study are included in the article/Supplementary material, further inquiries can be directed to the corresponding author.

## Supplementary material

SUPPLEMENTARY FIGURE S1 Overview of the experimental workflow for cultivated meat production using chicken-derived cell sources. (1) Cell Sources: Embryonated chicken eggs were used to isolate embryonic stem cells (ESCs), mesenchymal stem cells (MSCs), and muscle satellite cells. (2) Cell Differentiation: ESCs and satellite cells differentiated into myoblasts and further into myotubes (myogenesis), with downregulation of PAX7 and upregulation of MYMK. MSCs differentiated into adipocytes through adipogenesis, marked by increased expression of PPARG and FABP4. (3) Tissue Engineering Platforms: Differentiated cells were cultured in both 2D monolayer conditions and 3D systems such as spheroids and microcarriers. (4) Engineered Biomass Types: Muscle biomass was generated from myotubes, and fat biomass from adipocytes, both crosslinked with transglutaminase. A hybrid biomass was also engineered by combining muscle and adipocyte spheroids seeded onto microcarriers. (5) Application: The resulting constructs were proposed as food-grade, scalable, and sustainable cultivated meat with 10.63% protein content. Created with BioRender.com.

SUPPLEMENTARY FIGURE S2 Immunofluorescence staining of spheroids chicken myogenic and adipogenic cells cultured on microcarrier. Adipocytes derived from mesenchymal stem cell (MSC) spheroids were stained with HCS LipidTOX™ Red Neutral Lipid Stain, and Oil Red to detect intracellular lipid droplets (red). Staining confirmed adipogenic differentiation and lipid accumulation within 3D cultures on microcarriers. Myoblasts and myotubes derived from mesenchymal stem cell (MSC) spheroids were cultured on microcarriers to compose the cell biomass. Myogenic factor 5 (MYF5) and myosin heavy chain (MYHC) were detected using primary antibodies and Alexa Fluor 488-conjugated secondary antibodies (green), and nuclei were counterstained with DAPI (blue). Scale bar: 50 μm.

SUPPLEMENTARY VIDEO 1 Spontaneous contraction of in vitro differentiated chicken myotubes. Multinucleated myotubes derived from chicken myoblasts exhibit rhythmic contractile activity under standard differentiation conditions, indicating functional maturation of muscle fibers
