# Supplementary figures and images for: Establishment of chicken muscle and adipogenic cell cultures for cultivated meat production
Source: Front Nutr. 2025 Oct 13;12:1648935. doi: 10.3389/fnut.2025.1648935 (PMC12554555; doi:10.3389/fnut.2025.1648935)

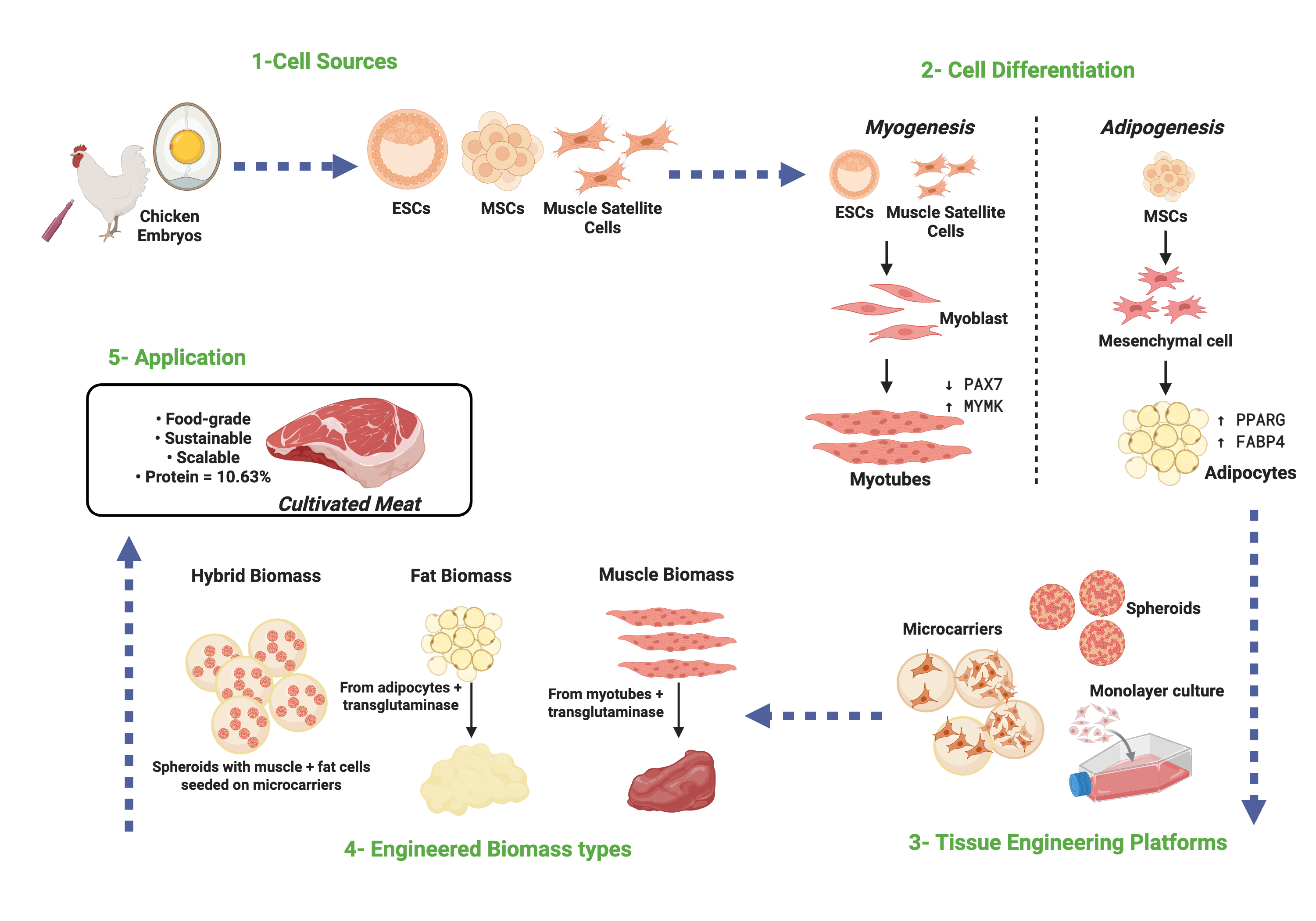

Supplement: SUPPLEMENTARY FIGURE S1 — Overview of the experimental workflow for cultivated meat production using chicken-derived cell sources. (1) Cell Sources: Embryonated chicken eggs were used to isolate embryonic stem cells (ESCs), mesenchymal stem cells (MSCs), and muscle satellite cells. (2) Cell Differentiation: ESCs and satellite cells differentiated into myoblasts and further into myotubes (myogenesis), with downregulation of PAX7 and upregulation of MYMK. MSCs differentiated into adipocytes through adipogenesis, marked by increased expression of PPARG and FABP4. (3) Tissue Engineering Platforms: Differentiated cells were cultured in both 2D monolayer conditions and 3D systems such as spheroids and microcarriers. (4) Engineered Biomass Types: Muscle biomass was generated from myotubes, and fat biomass from adipocytes, both crosslinked with transglutaminase. A hybrid biomass was also engineered by combining muscle and adipocyte spheroids seeded onto microcarriers. (5) Application: The resulting constructs were proposed as food-grade, scalable, and sustainable cultivated meat with 10.63% protein content. Created with BioRender.com. [file Image_1.JPEG]

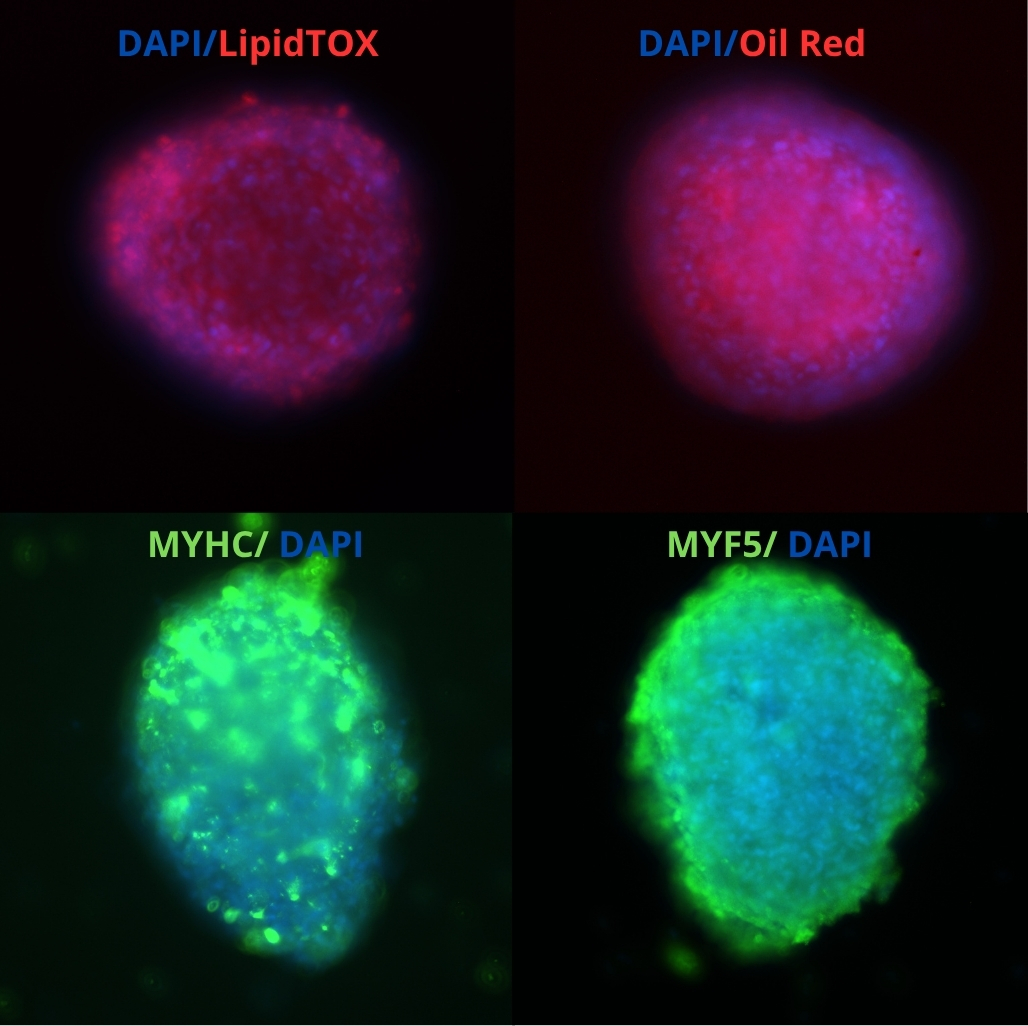

Supplement: SUPPLEMENTARY FIGURE S2 — Immunofluorescence staining of spheroids chicken myogenic and adipogenic cells cultured on microcarrier. Adipocytes derived from mesenchymal stem cell (MSC) spheroids were stained with HCS LipidTOX™ Red Neutral Lipid Stain, and Oil Red to detect intracellular lipid droplets (red). Staining confirmed adipogenic differentiation and lipid accumulation within 3D cultures on microcarriers. Myoblasts and myotubes derived from mesenchymal stem cell (MSC) spheroids were cultured on microcarriers to compose the cell biomass. Myogenic factor 5 (MYF5) and myosin heavy chain (MYHC) were detected using primary antibodies and Alexa Fluor 488-conjugated secondary antibodies (green), and nuclei were counterstained with DAPI (blue). Scale bar: 50 μm. [file Image_2.jpg]
