# Supplementary material for: Establishment of chicken muscle and adipogenic cell cultures for cultivated meat production
Source: Front Nutr. 2025 Oct 13;12:1648935. doi: 10.3389/fnut.2025.1648935 (PMC12554555; doi:10.3389/fnut.2025.1648935)
Supplement: Supplementary file 4 [file Table_1.docx]

**Supplementary Table S1:** Antibodies used for immunostaining.

^1^Used with Alexa Fluor 488 anti-mouse secondary antibody.

| **Antibodies*** | **Dilution** | **Catalog Number** |
| --- | --- | --- |
| ***Primary antibodies*** |  |  |
| mouse monoclonal anti-Pax7, Alexa Fluor 488 | 1:50 | Cat# SC-81648, Santa Cruz Biotechnology |
| mouse monoclonal anti-Myogenin (clone F5D)^1^ | 1:50 | Cat# SC-12732, Santa Cruz Biotechnology |
| mouse monoclonal anti-Desmin (clone D33)^1^ | 1:50 | Cat# MA5-13259, Thermo Fisher |
| mouse monoclonal anti-MyoD (clone 5.8A)^1^ | 1:100 | Cat# MA5-12902, Thermo Fisher |
| mouse monoclonal anti-Myosin 4 (clone MF20)^1^ | 1:100 | Cat# 14-6503-82, Thermo Fisher |
| rabbit polyclonal anti-Myf5^2^ | 1:100 | Cat# AB125301, Abcam |
| rabbit polyclonal anti-ITGA7^2^ | 1:100 | Cat# SL21333R, Sunlong Biotech |
| ***Secondary antibodies*** |  |  |
| Alexa Fluor 488 goat anti-mouse IgG (H+L) | 1:800 | Cat# A11029, Thermo Fisher |
| Alexa Fluor 488 goat anti-rabbit IgG (H+L) | 1:800 | Cat# A11034, Thermo Fisher |

^2^Used with Alexa Fluor 488 anti-rabbit secondary antibody.

*All antibodies were diluted in 1% BSA and 0.1% sodium azide.

**Supplementary Table S2:** *Gallus gallus* (Chicken) RT-qPCR primer sequences.

| **Gene** | **Forward primer (5′ → 3′)** | **Reverse primer (5′ → 3′)** | **Fragment (bp)** | **Reference** |
| --- | --- | --- | --- | --- |
| *cOCT4* | TGCAATGCAGAGCAAGTGCTGG | ACTGGGCTTCACACATTTGCGG | 114 | Giotis et al., 2019 |
| *cSOX3* | GTCGGGGTGGGCCAGAGGAT | GCTGTTCATGCCCGGGTGCT | 114 | Giotis et al., 2019 |
| *cNANOG* | TGCACACCAGGCTTACAGCAGTG | TGCTGGGTGTTGCAGCTTGTTC | 120 | Giotis et al., 2019 |
| *cSALL4* | GTCCACTGCGGACCCCAACG | GGTGGAGAAGGCACGGCCAC | 136 | Giotis et al., 2019 |
| *cCLDN3* | GGGTGGTTTCGGTCAGCGGG | GATGCTGCACAGCCAGCCCA | 116 | Giotis et al., 2019 |
| *cKIT* | AGCGAACTTCACCTTACCCG | CTGGGAATCCAGTTGCCACA | 181 | Han et al., 2018 |
| *cLIN28A* | CCGAGAATGAGTCCCAACCC | GGTGAATTCAACGGCTTCGC | 197 | Han et al., 2018 |
| *cPPARG* | GTGACCTTAATTGTCGCATCCA | GCATTCGCCCAAACCTGATG | 114 | Pasitka et al., 2022 |
| *cADIPOQ* | TGCTATTAAGGCCCCATCCTG | GCAACATCCAGGAGGCTACA | 122 | Pasitka et al., 2022 |
| *cPCK1* | TGGAGAGAATTCCCGTGTGC | TCCCACAGCTGCTCAACAAT | 505 | Pasitka et al., 2022 |
| *cADRP* | GACTTGCCCGGTATTCCATAAT | CTTCTTCCTGCTTCACTTCCTC | 93 | Pasitka et al., 2022 |
| *cFABP4* | GCCTGACAAAATGTGCGACC | TTCCTGGTAGCAAACCCCAC | 105 | Pasitka et al., 2022 |
| *cPAX7* | CAGTAGAGACAGGCCAAGC | GGAGTTGGGAAGGAGTAGGG | 135 | Hong & Do, 2024 |
| *cMYOD* | GCTCTCGCAGGAGAAACAG | CTGGAGGCAGTATGGGACAT | 159 | Hong & Do, 2024 |
| *cMYMK* | TGCGCTATGACATCCTGGAGTA | GGGACACCCAGATGGACAGA | 63 | Ju et al., 2023 |
| *cMYH1E* | TGGCACAGTGGACTACAACATCT | ACCATAGGTGGCAAACAGTAAGG | 127 | Ju et al., 2023 |
| *cCollagen I α1* | GTCCTGCTGGATTTGCTGG | GAAACCAGTAGCACCAGGG | 203 | Ma et al., 2024 |
| *cCollagen I α2* | TGATCCATCTAAAGCGGCTG | TTTGCCAGGGTGACCATCTT | 208 | Ma et al., 2024 |
| *cLaminin* | CGCGATTTCTGATTTTGCCG | CATTGCAGTCACAAGGCAAG | 212 | Ma et al., 2024 |
| *cFibronectin* | GTGCTACGACGATGGGAAAA | GCAGTTGACGTTGGTGTTTG | 220 | Ma et al., 2024 |
| *cElastin* | CTACTGGGACAGGTGTTGGA | CACCATAGGCTCCTGCCTT | 204 | Ma et al., 2024 |
| *MT-CYB Gallus gallus* | AGCAATTCCCTACATTGGACACA | GATGATAGTAATACCTGCGATTGC | 133 | Pasitka et al., 2022 |
| *cTBP* | TTGTGTCCACGGTGAATCTTG | TCGGGCACGAAGTGCAAT | 62 | Ju et al., 2023 |
